# Quasicrystals: What do we know? What do we want to know? What can we know?

**DOI:** 10.1107/S2053273317016540

**Published:** 2018-01-01

**Authors:** Walter Steurer

**Affiliations:** a Department of Materials, ETH Zurich, Leopold-Ruzicka-Weg 4, Zurich, 8093, Switzerland

**Keywords:** quasicrystals, structure analysis, higher-dimensional crystallography, stability of quasicrystals, quasicrystal growth

## Abstract

More than 35 years and 11 000 publications after the discovery of quasicrystals by Dan Shechtman, quite a bit is known about their occurrence, formation, stability, structures and physical properties. It has also been discovered that quasiperiodic self-assembly is not restricted to intermetallics, but can take place in systems on the meso- and macroscales. However, there are some blank areas, even in the centre of the big picture. For instance, it has still not been fully clarified whether quasicrystals are just entropy-stabilized high-temperature phases or whether they can be thermodynamically stable at 0 K as well. More studies are needed for developing a generally accepted model of quasicrystal growth. The state of the art of quasicrystal research is briefly reviewed and the main as-yet unanswered questions are addressed, as well as the experimental limitations to finding answers to them. The focus of this discussion is on quasicrystal structure analysis as well as on quasicrystal stability and growth mechanisms.

## Abstract 2

The state of the art of quasicrystal research is critically reviewed. Fundamental questions that are still unanswered are discussed and experimental limitations are considered.

## 1. Introduction   

Dan Shechtman was the first to identify a rapidly solidified intermetallic phase as a representative of a novel class of long-range-ordered (LRO) phases with icosahedral diffraction symmetry (Shechtman *et al.*, 1984 ▸). The term ‘quasicrystal’ for such intermetallics was coined by Dov Levine and Paul J. Steinhardt in an article published only a few weeks after Shechtman’s seminal paper (Levine & Steinhardt, 1984 ▸). This term refers to the class of ‘quasiperiodic functions’, which were introduced as such by the mathematician Harald Bohr (Bohr, 1924 ▸, 1925 ▸, and references therein). Amazingly, quasicrystals (QCs) were prepared in the course of studies of intermetallic phase diagrams unknowingly, long before Shechtman’s discovery (see, *e.g.*, Hardy & Silcock, 1956 ▸; Palenzona, 1971 ▸). Since, in most cases, only X-ray powder-diffraction methods were routinely used for sample characterization at that time, the fivefold diffraction symmetry characteristic of icosahedral QCs did not immediately catch the eye, unlike Shechtman’s electron diffraction patterns. Furthermore, it seems that nature created QCs aeons earlier. According to findings in meteorites, QCs may have been formed billions of years ago (see Bindi *et al.*, 2009 ▸, 2016 ▸, and references therein).

**Figure 1:**
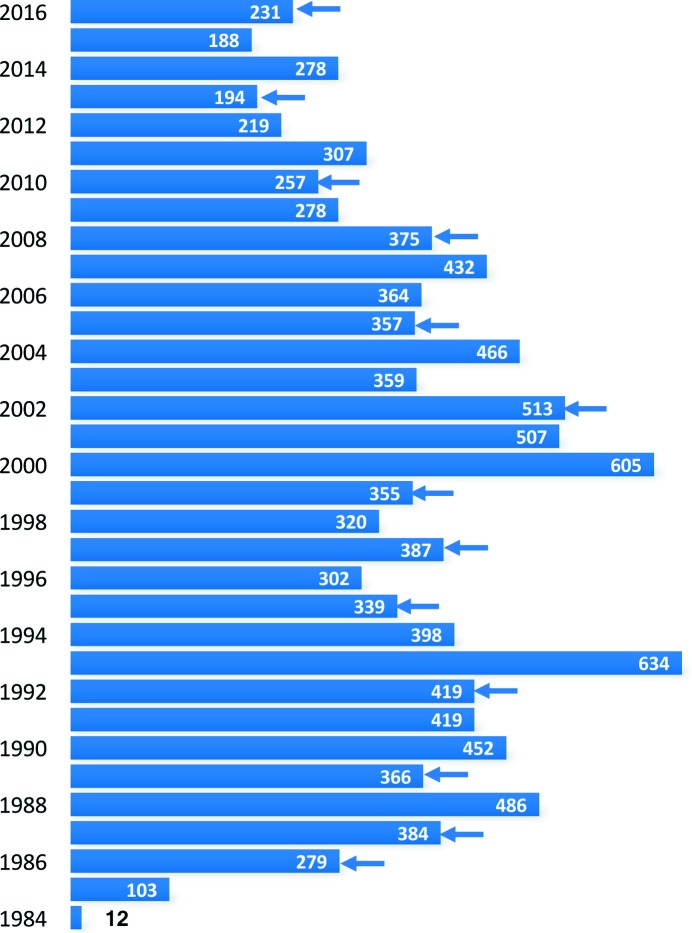
The number of articles on QCs in the years following the seminal publication by Shechtman *et al.* (1984 ▸). In some years the numbers peak owing to the publication of proceedings of international conferences on quasicrystals (ICQs) that took place in the year before. The general trend of the publication rate shows a decrease since the year 2000. This reflects the decreasing interest in, and funding for, QC research because of the lack of important applications.

What is the state of the art of quasicrystal research more than 35 years and 11 000 publications (see Fig. 1 ▸) after Dan Shechtman’s discovery? We know the structures of several decagonal and icosahedral QCs (DQCs and IQCs, respectively) almost as well as it is possible to know them and as well as we need to know them. The same is true for their physical properties, which do not differ significantly from those of high-order approximant crystals (ACs). ACs are built from the same structural subunits, frequently called ‘clusters’ (see also Steurer, 2006*a* ▸; Henley *et al.*, 2006 ▸; Jung & Steurer, 2011 ▸), but in a periodic way. In contrast, we do not know enough about the thermodynamic stability of QCs, and the way they form and grow from the melt. These are the main unanswered questions from my point of view.

Why is it worth investing more time and money in the pursuit of answers to these questions? What is so interesting about QCs that would justify the more than three decades of invested research and the resulting 11 000 publications so far, when potential applications seem to be quite limited (see, *e.g.*, Dubois, 2012 ▸, and references therein)? Well, it is simply the quasiperiodic structural LRO, which is fundamentally different to periodic order, which was long believed to be the only possible state for single-phase crystalline materials in thermodynamic equilibrium. The other two classes of aperiodic crystals, incommensurately modulated structures (IMSs) and composite or host–guest structures (CSs), which have been known for longer, are not as far away from average periodicity as QCs (see Steurer & Haibach, 1999 ▸; Deloudi & Steurer, 2012 ▸; Janssen *et al.*, 2007 ▸; and references therein). In contrast to QCs, IMSs and CSs can be considered as much better understood minor modifications or intergrowths of periodic structures.

In the following, the fundamental unanswered questions of quasicrystal research will be addressed with the focus on structure, stability and growth of intermetallic QCs. For reviews on these topics see, *e.g.*, the special issue on QCs of the Royal Society of Chemistry (2012 ▸). Finally, I want to emphasize that this topical review reflects just my personal view on the past, present and future of quasicrystal research.

## 2. Occurrence of quasicrystals   

**Figure 2:**
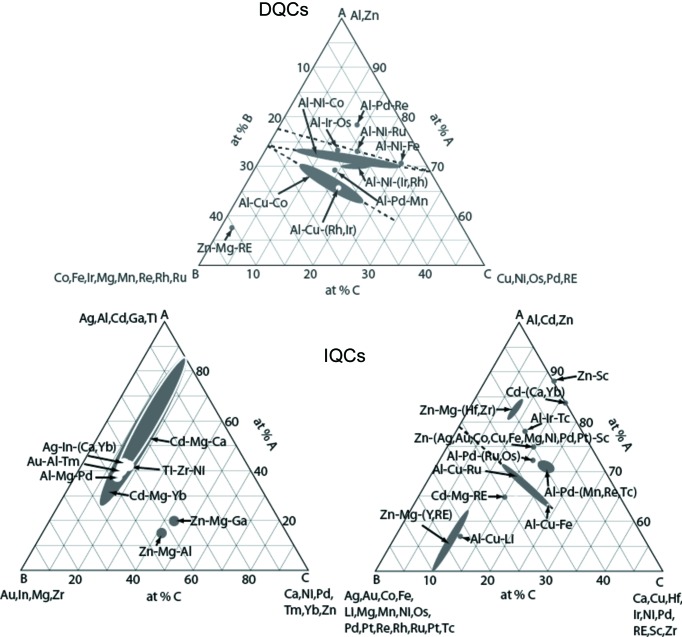
Concentration triangles showing the approximate compositional stability ranges of most DQCs (top) and IQCs (bottom). Each of the elements listed in parentheses can be a constituent of a specific QC. For instance, Cd–(Ca,Yb) represents only the QCs in the binary systems Cd–Ca and Cd–Yb. RE means rare-earth elements. Extended compositional stability ranges reflect the existence of intrinsic substitutional disorder. Most QCs, however, have narrow compositional stability ranges, *i.e.* they are line compounds.

QCs are binary or ternary intermetallic compounds, in many cases accompanied by low-order ACs with slightly different chemical compositions. So far, there is no known case of a quasicrystal transforming to an approximant crystal with exactly the same chemical composition, either as a function of temperature or with increasing pressure. Up to now, only QCs with icosahedral, pentagonal or decagonal symmetry have been found to be thermodynamically stable [Fig. 2 ▸; compare also Figs. 5.24 and 5.33 in Steurer & Dshemuchadse (2016 ▸)]. One hypothesis explaining the prevalence of fivefold symmetry in intermetallic QCs is based on cluster symmetry and packing possibilities. For instance, structural subunits with eight-, nine- or 12-fold rotational symmetry could be easily accommodated and packed in tetragonal, trigonal and hexagonal crystal structures, respectively. There is no need for potentially more efficient quasiperiodic packings that might be obtained by sacrificing the advantages of periodicity (phonons and Bloch waves are periodic). Indeed, there are only two dodecagonal QCs known so far. One, the telluride (Ta,V)_1.6_Te, is of rather poor quality and probably metastable (Krumeich *et al.*, 2012 ▸, and references therein). The other is a Mn-rich quaternary alloy, Mn_72.0−*x*_Cr_5.5+*x*_Ni_5.0_Si_17.5_ with *x* = 0 or 2.0, which seems to be more stable, but is also of low crystal quality (Iwami & Ishimasa, 2015 ▸). Sevenfold symmetry is very rare in intermetallic compounds but frequently found in some borides, which can be seen as ACs (Steurer, 2007 ▸; Orsini-Rosenberg & Steurer, 2011 ▸). However, no quasicrystal with sevenfold symmetry has been found so far. Potential QCs with 11-, 13- or 15-fold rotational symmetry could be energetically unfavourable for steric reasons. Indeed, structural subunits with such or higher symmetries are not currently known in intermetallics. Furthermore, quasiperiodic structures with these symmetries would have significantly lower degrees of average periodicity. This means that their atomic sites would show large deviations from their (in these cases badly defined) periodic average structures (Deloudi & Steurer, 2012 ▸), which can be of importance for the propagation of phonons and the formation of Bloch waves for electrons.

Self-assembly is quite different and much better understood in the case of mesoscopic and macroscopic quasiperiodic arrangements. Instead of chemical bonding, electrons and phonons, specific pair potentials, three-body interactions and the shape entropy play the decisive role there. In contrast to intermetallic QCs, 12-fold symmetry is the prevailing one in these cases (van Anders *et al.*, 2014 ▸; Barkan *et al.*, 2014 ▸; and references therein). Eighteen-fold symmetry has also been found for a specific experimental setup (Fischer *et al.*, 2011 ▸).

### 2.1. Open questions and challenges   

(i) What are the conditions (chemical composition, stoichiometry, valence electron concentration, temperature *etc*.) for the formation of stable QCs in intermetallic systems?

(ii) Can stable QCs with symmetries other than five-, eight-, ten- and 12-fold symmetries exist in intermetallics or in other chemical compounds?

(iii) We know that stable binary and ternary QCs exist. Do true quaternary and higher multinary QCs exist? Are unary QCs possible under extreme pressure (for instance, as quasi-binary electrides)?

(iv) Our goal is to predict the existence and stability of (all) possible QCs and their ACs based on our insight into their formation principles. There will be similar challenges and limitations for quasicrystal prediction as for the prediction of other complex intermetallics in general. One severe complication for quantum-mechanical calculations will be the missing periodic boundary conditions.

## 3. Stability of quasicrystals and approximants   

There has been a long discussion, which is still ongoing, about whether QCs are ‘energy- or entropy-stabilized’. In other words, whether quasiperiodic structural order can be a ground state of condensed matter (thermodynamically stable at 0 K) or has to be stabilized by entropic contributions from phonons, phasons and structural disorder. In the case of entropic stabilization, QCs would be high-temperature (HT) phases, only stable above a specific threshold temperature. In the early days of quasicrystal research, many QCs were discovered based on the working hypothesis of electronic stabilization by the Hume–Rothery mechanism (for a review see Tsai, 2003 ▸). Indeed, in several cases a pseudogap has been identified at the Fermi energy, originating either from Fermi-surface/pseudo-Brillouin zone nesting or from the hybridization between *d* and *p* states (see Lin & Corbett, 2007 ▸; Tamura *et al.*, 2004 ▸; Suchodolskis *et al.*, 2003 ▸; Mizutani, 2016 ▸; and references therein). The role of entropic contributions to the stabilization of QCs, in particular that of the phason modes, is still an ongoing matter of debate (de Boissieu, 2006 ▸, and references therein). Unfortunately, the mechanism for the stabilization of mesoscopic and macroscopic quasiperiodic arrangements cannot be directly transferred to intermetallic QCs because of the different interaction mechanisms.

At present, several experimental findings support possibility of a quasiperiodic ground state: (i) electronic stabilization *via* pseudogap formation at the Fermi energy; (ii) the high structural perfection of some QCs; (iii) the existence of periodic ACs.

What does the existence of periodic ACs have to do with a potential quasiperiodic ground state? Because of the need for periodic boundary conditions, quantum-mechanical (density functional theory, DFT) calculations are only possible on approximant crystal (AC) structures at present [for recent calculations see, *e.g.*, Mihalkovič *et al.* (2014 ▸) and references therein]. They do not show any structural instability at 0 K. By changing the chemical composition in smaller and smaller steps, one could go from low- to higher-order ACs and then, finally, to the quasicrystal (QC). This could support the hypothesis of QCs as a ground state of matter. Interestingly enough, at most only one or two stable low-order ACs have been experimentally observed in intermetallic systems featuring QCs so far. However, no devil’s staircase of ACs to a particular QC has been identified, as is known for IMSs (Bak, 1982 ▸, and references therein). This can be interpreted in such a way that once the chemical composition approximates that needed for the formation of a pseudogap at *E*_F_, the close-to-spherical pseudo-Brillouin zone of an IQC lowers the energy more than the less-symmetric cubic Brillouin zone of even a high-order AC. This would favour QC formation. Consequently, almost perfect QCs such as i-Cd–Yb or i-Zn–Mg–Sc may be stable at 0 K, while Al-based QCs with strong phasonic and chemical disorder may instead be HT phases.

### 3.1. Quasiperiodicity *versus* periodicity   

Why are almost all known crystal structures periodic and only a few quasiperiodic? In most cases, periodicity is the most efficient way of packing structural subunits (atoms, molecules, complex ions, clusters *etc*.) under the constraints of chemical bonding and structural dynamics. Phonons and Bloch waves are periodic as well. In contrast to amorphous structures, the Patterson (autocorrelation) function as well as the Fourier spectrum is discrete in both the periodic and quasiperiodic cases. This means that only an energetically favourable discrete set of interatomic distances exists. Furthermore, it has been shown that not only IMSs but also QCs have a kind of periodic average structure (Steurer & Haibach, 1999 ▸; Deloudi & Steurer, 2012 ▸).

The coexistence of both ACs and QCs in intermetallic systems proves that the common structural subunits (clusters) can principally arrange themselves equally well periodically and quasiperiodically, respectively. The decisive factor in the formation of one or the other is just a specific stoichiometry and, as a consequence, a valence-electron concentration favouring one or the other.

Structurally, a significant difference between ACs and QCs lies in the compatibility of clusters with non-crystallographic symmetry with the orientation of the flat atomic quasilattice layers [see, for instance, Fig. 9.9 in Steurer & Dshemuchadse (2016 ▸)]. The energetically favourable flat atomic layers can be seen as interfaces within complex structures; they also are the terminating layers forming the facets of crystals and quasicrystals.

### 3.2. Open questions and challenges   

(i) Can QCs be thermodynamically stable at 0 K? An experimental limitation for studying the low-temperature (LT) stability of QCs lies in the fact that the diffusion necessary for a transformation of quasiperiodic structures into periodic ones would be too sluggish at low temperatures.

(ii) For studying quasicrystal stability theoretically, one could perform first-principles calculations on a series of higher and higher ACs, 0/1, 1/1, 2/1, 3/2,…, *n*/*m* (where *m* and *n* are sequential Fibonacci numbers) (see, *e.g.*, Krajčí & Hafner, 2005 ▸). The results could explain why only low-order ACs have been found experimentally so far. The calculations should also allow one to extrapolate the findings to the stability of the corresponding QC (see also Steurer, 2012 ▸, and references therein).

## 4. Structure of quasicrystals   

Why are we interested in the analysis of crystal structures at all? At present, the main structural databases contain more than 1 200 000 entries for periodic crystal structures [>188 000 inorganics/intermetallics in the Inorganic Crystal Structure Database (ICSD); >875 000 organics/metalorganics in the Cambridge Structural Database (CSD); >107 000 proteins in the RCSB Protein Data Bank (PDB)] and 145 of incommensurate structures in the Bilbao Incommensurate Structures Database (B-IncStrDB). The number of quantitatively determined QC structures is much smaller (≃20) than the number of known stable QCs (≃50), but this number includes representatives of all QC structure types known to date (Steurer & Deloudi, 2009 ▸, and references therein). For examples of state-of-the-art *n*-dimensional (*n*D) structure analyses of IQCs and DQCs see, for instance, Takakura *et al.* (2007 ▸) and Logvinovich *et al.* (2014 ▸), respectively; for tiling-based analyses of DQCs at ambient and high temperatures see Kuczera *et al.* (2012 ▸) and (2014 ▸), respectively.

What is the added value of a (sometimes quite tedious) structure analysis of a QC? In most cases, it just adds to our understanding of the chemical-composition/crystal-structure relationship (see, *e.g.*, Steurer & Dshemuchadse, 2016 ▸). In only a few selected cases where physical properties have been studied as well does it improve our understanding of the chemical-composition/crystal-structure/physical-properties relationships. This information brings us closer towards one of the ultimate goals of materials design: from the desired physical/chemical properties of a material, find its chemical composition and the protocol for preparing it. In the case of QCs most of our goals are at present more basic: first, we want to understand the governing factors for the evolution of quasiperiodic order for a chemical compound of a given composition; in other words, the reason why a structure becomes quasiperiodic (see, *e.g.*, Steurer, 2004 ▸, 2012 ▸). Here it should be mentioned that the structural perfection of QCs can be comparable with that of periodic complex intermetallics in general. Consequently, dynamical-diffraction phenomena such as multiple diffraction are quite common; anomalous transmission effects have also been observed (see, *e.g.*, Kycia *et al.*, 1993 ▸). This indicates a high perfection of at least the quasiperiodic average structure, without saying anything about the possible deviations from it.

QC structure analysis is still far from being a straightforward task, although the higher-dimensional (*n*D) approach provides a powerful tool. It should be borne in mind that for a single specific QC structure in the *n*D description, there exists an infinite number of slightly different, but locally isomorphous, realizations in three-dimensional physical space. This means that the structures of actual quasicrystallites, taken from a larger single-crystalline QC, may not be congruent to each other, but all of them could be related to one and the same idealized *n*D structure model. In other words, we do not know *a priori* which of the infinitely many three-dimensional realizations of an *n*D structure model applies to a specific QC with known *n*D structure. However, we do not need to know this in order to understand the structural building principles and physical properties. If one needs to know more about the actual local structure of a QC, electron microscopy would be the method of choice.

### 4.1. Diffraction   

The characteristics (and definition) of all ideal crystals, be they periodic or aperiodic, is their pure-point Fourier spectrum 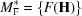, with 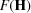 the structure factor (Fourier coefficient) for the diffraction vector **H**. This means for a diffraction experiment on a *d*-dimensional (*d*D) crystal that all its Bragg reflections have to correspond to δ peaks (within the experimental resolution) supported on a 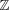 module (an additive Abelian group)
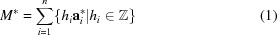
of rank *n* (*n* ≥ *d*) with reciprocal-basis vectors 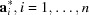. In the *n*D approach, *n* determines the dimension of the embedding space and *d* that of the actual (a)periodic crystal (usually *d* = 3) in physical space, which is also called parallel space (par-space), 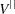. In practice, our first assumption is usually that our experimentally measured reflection intensities conform to Bragg reflections. This automatically implies periodicity of the *n*D structure model to be derived and applicability of the *n*D approach. This implication is not critical for three-dimensional on-average periodic structures, because any deviation from periodicity could be easily detected by imaging methods such as electron microscopy, for instance. In the case of on-average non-periodic structures, however, it would be more difficult to identify the kind of average LRO and the actual deviations from it.

Once the experimental data are accepted as Bragg reflections, the symmetry and metrics of the *n*D lattice can be determined in a straightforward way. For *n* = 3, this already determines the LRO of a structure (Bravais lattice). In contrast, for *n* > 3, the LRO of the three-dimensional quasiperiodic structure not only depends on the *n*D Bravais lattice type but also on the content of the *n*D unit cell, *i.e.* on the location, shape, partition and size of the *n*D hyperatoms. An *n*D hyperatom has a three-dimensional par-space component, which gives the atom in its usual three-dimensional description when intersected with the physical space. In the (*n* − 3)D perpendicular space (perp-space), 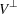, it is represented by an extended polyhedral distribution function called an occupation domain or atomic surface. This means that the kind of LRO of a quasiperiodic structure is not only coded in the *n*D Bravais lattice type but also in the position, size and shape of the atomic surfaces.

Consequently, determining the structure of a QC does not only mean identifying the positions **r**_*k*_ of the *m* *n*D hyperatoms in the *n*D unit cell, but also their components in the two subspaces of the *n*D embedding space 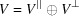, *i.e.* the three-dimensional atoms in 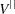 and the (*n* − 3)D atomic surfaces in 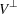. The structure factor is expressed as
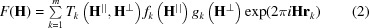
where 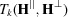 are the components of the atomic displacement parameters (ADPs) in par- and perp-space (phason factor, perp-space ADP), respectively, 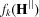 is the conventional atomic form factor, and 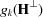 is the geometrical form factor, *i.e.* the Fourier transform of the atomic surface,
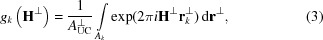
where 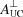 is the volume of the *n*D unit cell projected onto 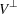, and 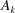 is the volume of the *k*-th atomic surface.

*Example*. The well known Penrose tiling (PT) is a special case of the infinite number of generalized PTs (Pavlovitch & Kléman, 1987 ▸), which not only differ in their LRO, but also in their number of different vertex environments (short-range order, SRO). Based on the limited amount of experimental Bragg reflection data that we can collect, we are not able to distinguish between all the different possible generalized PTs (see Chodyń *et al.*, 2016 ▸), let alone all the other different tilings that may underlie a given QC structure (see, *e.g.*, Deloudi *et al.*, 2011 ▸, and references therein), which all can have the same *n*D Bravais lattice and lattice parameters (see also Niizeki, 2004 ▸, and references therein).

The structures of ACs, which can all be determined by standard methods in a straightforward way, are very valuable for understanding the local structure of QCs (see, *e.g.*, Gómez & Lidin, 2003 ▸). They allow us to unambiguously identify the basic structural subunits (clusters) and show how they are connected (chemical bonding). Unfortunately, higher approximants are rare and not every QC is accompanied by one or more ACs. The best way to solve a QC structure at present is by a combination of *n*D charge flipping and the *n*D low-density elimination approach (see, *e.g.*, Palatinus *et al.*, 2011 ▸; Fleischer *et al.*, 2010 ▸; and references therein), which are direct-space phasing methods. The resulting electron-density distribution function provides the necessary information for designing a starting model for subsequent structure refinements.

One has to bear in mind that the result of an *n*D structure analysis is always an on-average strictly quasiperiodic structure, even if the actual structure is strongly disordered (see, *e.g.*, Ors *et al.*, 2014 ▸, and references therein) or just a twinned high-order AC (*cf*. Estermann *et al.*, 1994 ▸). This follows from the basic underlying assumption that the experimental intensity data can be interpreted as Bragg reflections. In order to get a realistic picture, diffraction-based QC structure analyses should always be complemented by imaging methods.

In the course of a quantitative structure refinement, an initial structure model is modified to describe the experimental diffraction data in the best possible way. In a standard three-dimensional structure refinement, only three-dimensional atomic models are fitted. This allows, rather easily, the identification of missing or misplaced atoms, unphysical atomic arrangements *etc*. In contrast, in an *n*D structure refinement, complex atomic surfaces and their partitioning have to be modelled. Consequently, a rather small number of parameters may have large implications for the LRO of the refined structure model in physical space. Furthermore, the total weight in the refinements of the many weak reflections with large perp-space components of the diffraction vectors, 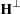, is usually small owing to the effect of the perp-space ADPs, 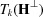, which decreases their calculated intensities drastically. This makes LRO models somewhat less reliable.

### 4.2. Electron microscopy   

Imaging methods are essential for the derivation and confirmation of proper structure models of QCs (see, *e.g.*, Deloudi *et al.*, 2011 ▸, and references therein). In particular, spherical-aberration- (*C*_s_-) corrected high-angle annular dark-field and annular bright-field scanning transmission electron microscopy (HAADF-STEM and ABF-STEM, respectively) in combination with energy-dispersive X-ray spectroscopy (EDX) and electron energy-loss spectroscopy (EELS), respectively, have proven to be a powerful tool for atomic resolution structure analysis, also allowing the identification of atomic species (see, *e.g.*, Yasuhara & Hiraga, 2015 ▸).

However, one has to bear in mind that electron micrographs show only projected structures, averaged over the sample thickness of ≃10 nm (≃50 atomic layers). This is no problem in the case of DQCs with a period of two to six atomic layers along the tenfold axis. It even makes it easier to identify the basic clusters and the ways they overlap. In the case of IQCs, the interpretation of the electron micrographs gets more difficult because of their three-dimensional quasiperiodicity. However, if a parallel set of flat atomic layers (quasilattice planes) is oriented exactly parallel or perpendicular to the incident electron beam, then the electron micrographs show rather easily interpretable structural features. For an objective method for the identification of the LRO of clusters from high-resolution transmission electron microscopy (HRTEM) images see Joseph *et al.* (1997 ▸).

### 4.3. Open questions and challenges   

(i) It is absolutely crucial for any kind of experimental structural study that the samples are well defined and characterized. This means, in particular, that they must have a well defined thermal history; in the best case, they should be in thermodynamic equilibrium. For intermetallic QCs, this would be possible only in the case of *in situ* HT measurements because of the sluggish diffusion and equilibration at lower temperatures. The usual compromise is to study samples quenched from a thermodynamic equilibrium HT state. If only as-cast samples were investigated, one could not distinguish the intrinsic structural and physical properties from features caused by chemical inhomogeneities, thermal gradients or incomplete structural transitions.

(ii) An important question concerns the role of clusters. Are they just a means of providing a clear and illustrative description of complex structures, or are they also energetically favourable subunits that are crucial for AC and QC structure formation? An interesting example is the DQC in the system Zn–Mg–Dy (Ors *et al.*, 2014 ▸), where clusters seem to be absent. Is this owing to disorder or an intrinsic structural feature?

(iii) It will not be possible to identify the LRO of even strictly quasiperiodic structures wth the same certainty as for periodic structures. While the number of *n*D Bravais lattices for DQCs and IQCs is limited, that of different two-dimensional and three-dimensional quasilattices (tilings underlying quasiperiodic structures) is unlimited.

(iv) The accurate determination of the atomic surfaces is limited by the comparably low perp-space resolution of diffraction experiments. Because of their complex shapes, the Bragg reflections with large |**H** ^⊥^| components are intrinsically weak and difficult to measure with good counting statistics. Furthermore, random phason fluctuations weaken these reflections even more. This has consequences for the determination of the LRO. A dynamic intensity range 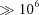, which can be achieved with standard charge-coupled device (CCD) detectors and synchrotron radiation at present, would be the minimum for a reliable structure analysis [see Fig. 1 of Weber *et al.* (2008 ▸), for instance]. Even for an extraordinarily large dynamic range of 10^9^, in i-Al–Cu–Fe, for instance, only reflections that could be related to a 13/8 AC with a lattice parameter of ≃130 Å (*i.e.* just a few cluster diameters) could be observed (Weber *et al.*, 2008 ▸).

(v) Only a combined diffraction/electron microscopy approach could identify limit-periodic structures, for instance, those with fractal atomic surfaces, and distinguish them from QCs (see, *e.g.*, Fujita & Niizeki, 2006 ▸; Niizeki, 2007 ▸).

(vi) Since the set of Bragg reflections for a QC is dense (‘essentially discrete’), multiple diffraction can lead to a significant bias in data collection. In particular, this has implications for the weak Bragg reflections with large |**H** ^⊥^| components, which are important for the accurate determination of the shapes of the atomic surfaces and consequently the LRO. The data could be corrected for this effect if several data sets were collected at different X-ray wavelengths. Each data set would suffer from different dynamic effects and a corrected data set could be derived.

(vii) In standard structure analysis, both observed and unobserved reflections have to be included in a structure refinement. However, since ideal QCs have infinitely many reflections in a given par-space range, not all unobserved reflections can be included. Only those reflections with calculated intensities within the dynamic range of the diffraction experiment should be considered and included in the refinements. This would greatly improve the reliability of refined model structures and perp-space ADPs (see Weber *et al.*, 2008 ▸).

(viii) The final step in a structure refinement should be the calculation of par-space maximum-entropy maps (which are free from truncation effects) of the electron-density distribution function to identify shortcomings of the refined model structure (see, *e.g.*, Steurer *et al.*, 1993 ▸). Thereby the phases of the less well determined weak reflections should be allowed to vary.

### 4.4. Surface structure analysis   

The surfaces of QCs and their approximants do not reconstruct as far as we know (see, *e.g.*, Ledieu & Fournée, 2014 ▸; Hars *et al.*, 2016 ▸). Consequently, knowledge of their structures as obtained by scanning tunnelling microscopy (STM) or atomic force microscopy (AFM) can be very helpful for checking three-dimensional structure models obtained by diffraction methods (see, *e.g.*, Papadopolos *et al.*, 2008 ▸). The terrace structure of QCs that were cut slightly tilted with respect to a facet (flat atomic layer) reflects the distances between quasilattice planes. It also shows that the clusters in IQCs are not stable subunits that maintain their shapes at the surface, as suggested a while ago (Rösch & Trebin, 2008 ▸). In contrast, the flat surface runs through the clusters along flat atomic layers (Jung & Steurer, 2011 ▸, and references therein).

### 4.5. The Penrose tiling – a proper quasilattice model for real QCs?   

There are an infinite number of different quasiperiodic tilings. Why are the two-dimensional Penrose tiling and the three-dimensional Ammann tiling (also called the three-dimensional Penrose tiling) passable models for the cluster-decorated quasilattices underlying both DQCs and IQCs in so many cases?

(*a*) The proper projection of a five-dimensional hypercubic unit cell onto two-dimensional space gives a decagonal structural subunit that can be related to the basic clusters that are actually observed.

(*b*) The proper projection of a six-dimensional hypercubic unit cell onto three-dimensional space gives an endohedral, triacontahedral structural subunit that can be related to the basic clusters that are actually observed.

(*c*) The best arrangement of these structural subunits [‘quasi-unit cells’ according to Steinhardt *et al.* (1998 ▸)] corresponds to the packing of the respective *n*D hypercubic unit cells, *i.e.* to the *n*D lattice (Steurer & Deloudi, 2012 ▸). The quasi-unit cells correspond to covering clusters of the PT (Gummelt clusters) and partially overlap in a specific way.

These findings explain why the *n*D approach works so well for QCs. It is the correspondence of the packing principles of *n*D unit cells (without overlaps) in a hyperlattice and *d*D clusters (quasi-unit cells, with overlaps). The two-dimensional/three-dimensional quasi-Bravais lattices of two-dimensional/three-dimensional quasi-unit cells can be considered as the counterparts to two-dimensional/three-dimensional Bravais lattices. There are a limited number of decagonal and icosahedral quasi-Bravais lattices corresponding to just one (*P*) five-dimensional decagonal and three (*P*, *I*, *F*) six-dimensional icosahedral Bravias lattices (Rokhsar *et al.*, 1987 ▸). The actual QC structures result from a specific hyperatom decoration of the quasi-unit cells. It is crucial to identify the correct *n*D lattice parameters, which determine the cluster diameters in physical space. Because of scaling symmetry, this is not a straightforward task [*cf*. Fig. 8 of Steurer *et al.* (1993 ▸)]. Since the scaling symmetry only refers to the positions of atoms and not to their kind, the strongest peaks on a Patterson map will mark the intercluster vectors (Cervellino *et al.*, 1998 ▸).

### 4.6. Structural complexity – quasiperiodic *versus* periodic complex intermetallics   

How complex are QC structures? They do not have a three-dimensional unit cell – does this mean that they are even more complex than the most complex periodic intermetallics? For instance, is the periodic structure of Al_55.4_Cu_5.4_Ta_39.1_, with 23 256 atoms per cubic face-centred unit cell and with 1393 parameters refined against 48 023 unique reflections (Weber *et al.*, 2009 ▸; Conrad *et al.*, 2009 ▸) more or less complex than that of a QC such as i-Cd_5.7_Yb, with 251 parameters refined against 5024 unique reflections (Takakura *et al.*, 2007 ▸)? Or, how complex are compositionally complex structures such as high-entropy alloys in comparison (see, *e.g.*, Kozak *et al.*, 2015 ▸, and references therein)?

First of all, we have to clarify what structural complexity means, and how it can be described and quantified. Second, why is it of interest at all to deal with these questions? The term ‘complex’ is frequently used just for labelling intermetallic structures with specific features like large unit cells, or cluster structures with different length scales and chemical-bonding properties, or unusual ordering phenomena *etc*. (see also Dshemuchadse & Steurer, 2013 ▸, and references therein). In many cases these structural particularities can give rise to the unusual physical properties of intermetallics such as low thermal/electronic conductivity. Understanding the principles of their formation can pave the way to designing novel complex structures with interesting physical properties. Therefore, it may be of interest to discuss what structural complexity could mean, how it could be quantified (see, *e.g.*, Estevez-Rams & González-Férez, 2009 ▸), and how periodic and quasiperiodic structures could be compared.

Structural complexity is not necessarily related to a large number of free (variable) structural parameters. For instance, a complex coordination polyhedron (cluster shell) such as a truncated cuboctahedron with 48 vertices can be described by just three fixed positional parameters in the general Wyckoff position of the cubic space group 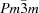. If the same truncated cuboctahedron is slightly distorted in an irregular manner, then it would be described in the triclinic space group *P*1, with 3 × 48 positional parameters. Does that mean that its structure would be much more complex now? It depends on the definition of complexity, as we will see below. What role does symmetry play? There is just one symmetry operator in *P*1 but there are 48 in 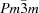. It should be kept in mind that structural symmetry results *a posteriori* from the specific way the atoms self-assemble and not the other way around.

There exist several ways to describe the complexity of a system. Which is the most appropriate one for crystal structures with different kinds of LRO, *i.e.* periodic and aperiodic ones? We will discuss just two approaches, taking into account that the structure-determining factors are mainly geometrical packing principles under the constraints of chemical bonding. The resulting complexity is reflected in the number of atoms per unit cell, if there is any, reduced in some way by symmetry. A pragmatic way to quantify complexity can be based on the amount of information necessary to describe a structure or its growth in a useful way. By ‘useful’ we mean that there is no need to know the actual position of each vacancy or dis­ordered atom in a crystal, for instance; structural disorder can be described sufficiently well by statistical parameters.

#### 4.6.1. Algorithmic complexity   

This way of quantifying the complexity of a system is related to the minimum size of an algorithm needed for its full description. In the case of a periodic crystal structure, the additive algorithmic complexity for a structure *s* with *N* atoms,
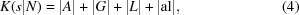
consists of |*A*|, the set of atomic coordinates in the asymmetric unit, |*G*|, the generators of the space group, |*L*|, the set of lattice basic vectors, and |al|, the algorithm needed to generate the full crystal structure by multiple (up to infinite) iteration (Estevez-Rams & González-Férez, 2009 ▸). In the case of a quasiperiodic structure we have to replace |*A*|, the set of atomic coordinates in the asymmetric unit of the *n*D unit cell, by |*AS*|, the set of parameters fully describing the (*n* − 3)D atomic surfaces, *i.e.* their coordinates, shapes, partitioning and chemical occupancies. Furthermore, an algorithm is needed to produce the three-dimensional quasiperiodic crystal structure out of the *n*D structure along its intersection with the physical space. This step will be the most laborious one.

Our example of a truncated cuboctahedron with 48 vertices would have the same algorithmic complexity in regular and distorted form. In the cubic space group 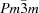 we need 48 symmetry operators to generate all 48 vertices starting from a single coordinate triplet. In the triclinic space group *P*1 all 48 coordinate triplets are already explicitly given. Intuitively, however, one may say that the loss of symmetry would make a system more complex.

The algorithmic complexity of large biomolecular structures, which can have hundreds of thousands of atoms per unit cell, is certainly higher than that of QCs.

#### 4.6.2. Symbolic complexity   

Symbolic complexity is related to the number of different structure motifs (atomic environment types, AETs) as a function of the system size *R*. The symbolic complexity of a structure is reflected in its *R* atlas.

A periodic lattice has just one set of vertex configurations, while the PT, for instance, has eight different ones. If there exists a patch (a set of atoms such as a covering cluster or a unit cell, for instance) from an ordered structure that has exactly the same coordination by copies of this patch everywhere in the infinite structure then the structure is periodic. If there exist only patches that have a finite number (>1) of different local arrangements of copies of these patches, then such an ordered structure is quasiperiodic or non-periodic in a more general way. From this point of view, quasiperiodic structures would be more complex than periodic ones. Furthermore, one could also compare the complexity of the patches themselves.

Our example of the truncated cuboctahedron with 48 vertices would have different symbolic complexities in its regular and in its distorted form. In the case of the regular polyhedron, each vertex would be surrounded by a square, a regular hexagon and a regular octagon. So, all vertices would have the same environment. This is different for an arbitrarily distorted truncated cuboctahedron. All vertex environments would still consist of quadrangles, hexagons and octagons, but all of them would be irregularly distorted. Consequently, the distorted polyhedron would have 48 different vertex configurations, and would be more complex than the regular one.

If one were to compare the *R* atlases of the most complex biomolecules with those of QCs, then biomolecules would certainly be found to be more complex owing to the large number of atoms (and their AETs) per unit cell.

#### 4.6.3. Relative complexity of aperiodic crystals in general and the *n*D approach   

Let us start from a simple periodic structure (PS) with period *a* and apply a displacive sinusoidal modulation with incommensurate period α*a*, with α an irrational number. This immediately changes the period of the structure from *a* to infinity. However, the algorithmic complexity of the incommensurately modulated structure is only slightly higher than that of the PS, although the unit cell now formally contains an infinite number of atoms. In the case of a commensurate modulation, we can continuously increase the number of atoms per supercell and, consequently, the symbolic complexity. Since we can always describe the respective modulated structure by the *n*D approach in the same way, changing the superperiod (modulation wavelength) by changing the slope of the cutting (physical) space would not change the algorithmic complexity of the structure.

Similar considerations apply to ACs and QCs. The algorithmic and symbolic complexities of three-dimensional and *n*D periodic structures can be compared with each other if the three-dimensional structures can be embedded as approximants in *n*D space. Then, a small perp-space shear of an *n*D structure can lead either to an AC or a QC. This would result in equal algorithmic complexities of ACs and QCs. This again is counterintuitive, because low- and high-order ACs with small and very large unit cells, respectively, would also have the same algorithmic complexity but very different symbolic complexities.

**Figure 3:**
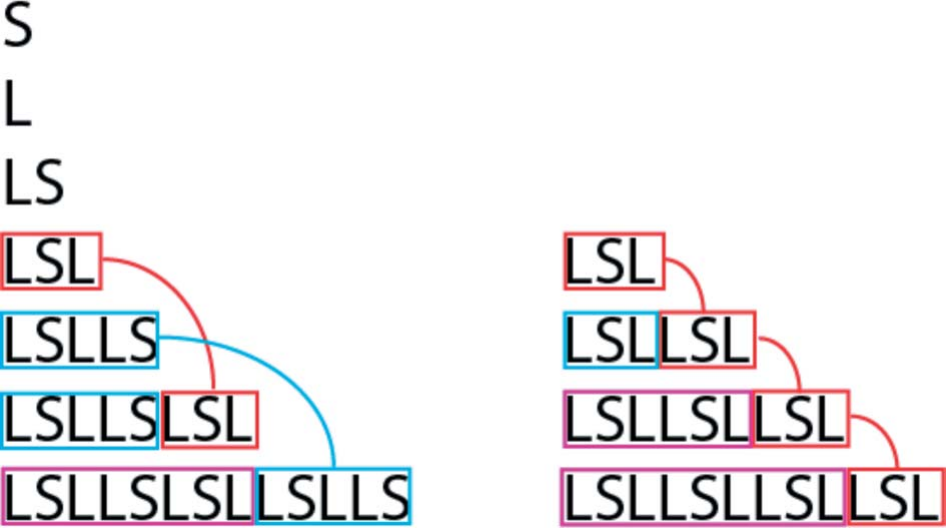
The Fibonacci sequence (left) and its approximant (right) generated by the recursive concatenation operations *w*_*n*+2_ = *w*_*n*+1_ + *w*_*n*_ and *w*_*n*+2_ = *w*_*n*+1_ + *w*, respectively.

How does this work if we stay in par-space? Let us check this by using the example of the Fibonacci sequence (FS) and one of its ACs. The generating algorithms differ slightly from one another because the AC is generated by unit-cell translations, *w*_*n*+2_ = *w*_*n*+1_ + *w*, with the unit cell (word) *w* = LSL, for instance, and the FS by the concatenation *w*_*n*+2_ = *w*_*n*+1_ + *w*_*n*_, with the initial conditions *w*_0_ = S, *w*_1_ = L. Consequently, the algorithmic complexity would be equal as well (Fig. 3 ▸).

For calculating the symbolic complexity, we would choose the patch (word) LSL of the FS and check the surrounding of every word in the infinite AC. It would always be the same. In the case of the FS, we would have more than one type of surrounding such as LSL**LSL**SLL, LLS**LSL**LSL, LSL**LSL**LSL. Consequently, the symbolic complexity of the FS would clearly be higher.

## 5. Quasicrystal growth   

‘*For this reason, I was somewhat doubtful that nature would actually produce such ‘quasi-crystalline’ structures spontaneously*’ said Roger Penrose (Thomas, 2011 ▸). ‘*I couldn’t see how nature could do it because the assembly requires non-local knowledge*’. The growth of quasiperiodic structures is still not fully clarified; however, some realistic models and simulations have been published recently (see Kuczera & Steurer, 2015 ▸, and references therein). It should be borne in mind that no atomistic growth models exist for complex intermetallics either. How does the 1000th atom find its site in a giant unit cell with thousands of atoms? When does a structure grow periodically, when quasiperiodically?

The driving forces for the growth of intermetallic QCs are: (*a*) a well defined chemical composition providing a specific valence-electron concentration; (*b*) energetically favourable structural subunits (clusters) with well defined ‘overlap’ rules and compositions differing from the overall composition – the SRO component; (*c*) minimum fluctuations of the local composition (MFLC) and, in consequence, of the chemical potential, around the given global average one; and (*d*) faceted growth [low-energy atomic layers (see, *e.g.*, Steurer, 2011 ▸; Dshemuchadse *et al.*, 2011 ▸; Katz & Duneau, 1986 ▸)] – the LRO component.

**Figure 4:**
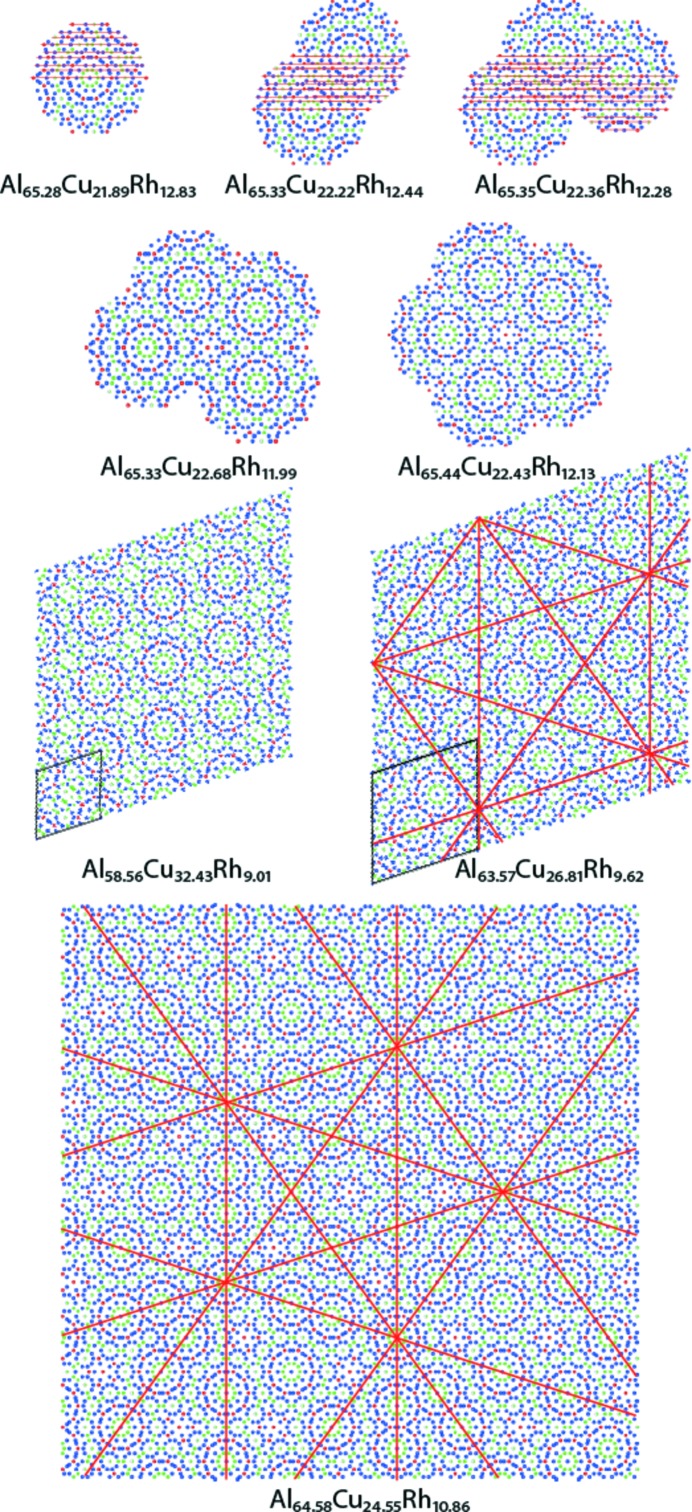
Arrangements of columnar clusters forming decagonal Al–Cu–Rh (Kuczera *et al.*, 2012 ▸). Their chemical composition varies with increasing size. The three structures at the bottom are patches of two periodic and one quasiperiodic arrangement of clusters. They differ significantly in chemical composition and symmetry as indicated by the orange lines. Obeying the overlap rules also means obeying the FALC rule, which is demonstrated with a subset of layer lines on the topmost clusters.

**Figure 5:**
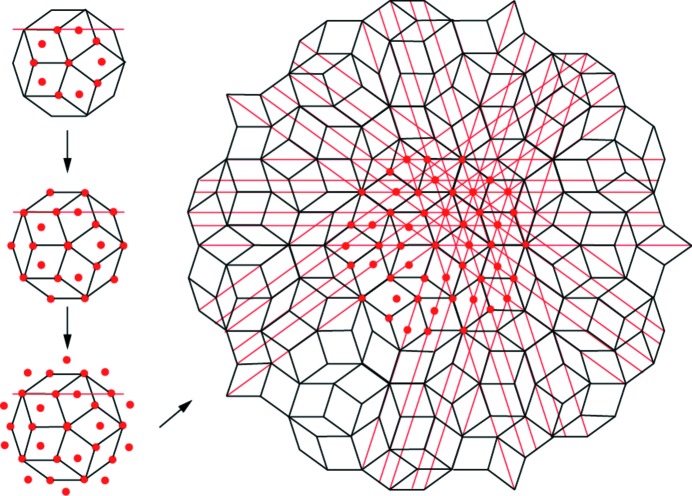
Example of a schematic growth algorithm. In the first step one cluster (marked by a red dot in the centre of the cluster at top left) is surrounded by ten others obeying the overlap rules. Then a ring of ten more clusters is added, and so on. The red dots mark cluster centres, which all lie on intersections of quasilattice lines. For creating a quasiperiodic structure the FALC and the MFLC rule must be obeyed.

**Figure 6:**
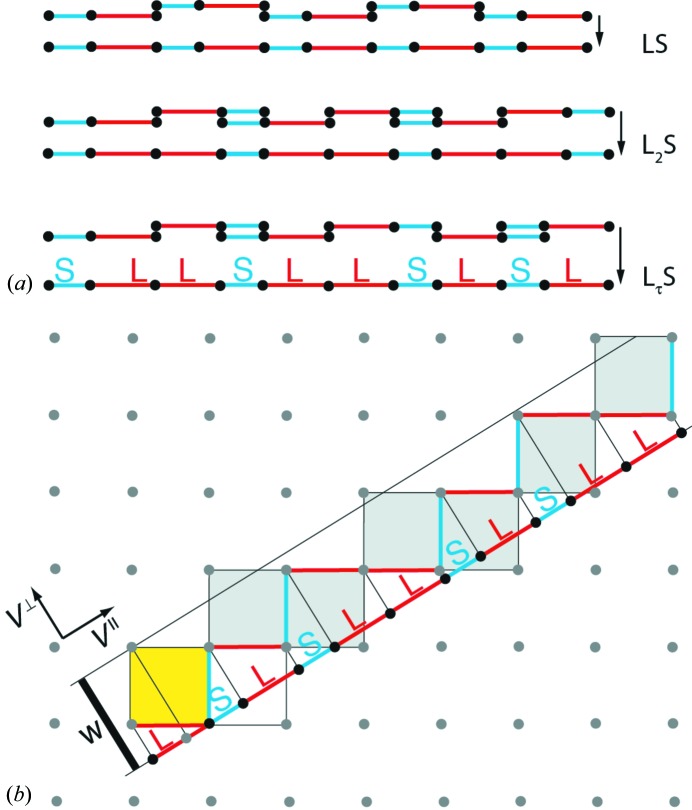
(*a*) Sequences with overall composition LS (top), L_2_S (middle) and L_τ_S, *i.e.* the FS (bottom), with the decomposition into covering clusters of the type (LS) in both orientations. The upper two sequences are rational approximants of the quasiperiodic FS. (*b*) The overall stoichiometry of the FS, *i.e.* the ratio of the number of L’s to the number of S’s, is defined by the slope of the strip. Its width W constrains the maximum stoichiometry fluctuations around the ideal value τ^−1^.

As indicated in Fig. 4 ▸, the main factors controlling AC/QC growth are the local chemical composition(s) of the fundamental cluster(s) *versus* the global composition and the flat atomic layers continuation (FALC) rule [Fig. 5 ▸; see also Figs. 2 and 3 of Steurer & Deloudi (2014 ▸)]. The MFLC rule will lead to quasiperiodic growth (Fig. 6 ▸) if the the stoichiometry allows it. Local phason flips of clusters do not change the chemical composition; however, they break the FALC rule. In contrast, strongly phason-strained structures, which are in the extreme case orientationally twinned nanodomain structures (Honal *et al.*, 1998 ▸, and references therein), will have an AC chemical composition. Such structures can be seen as textured polycrystalline structures of ACs rather than as disordered quasiperiodic structures.

### 5.1. Open questions and challenges   

(i) We need to continue developing realistic three-dimensional growth models, *i.e.* starting with nucleation and growth from the melt (see, *e.g.*, Kuczera & Steurer, 2015 ▸). These should take into account that chemical SRO already takes place in the melt close to the melting temperature. Depending on the stoichiometry either ACs or QCs should grow.

(ii) We also need to study the heterogeneous growth of QCs on surfaces of periodic crystals by AFM/STM; see, for instance, the growth of decagonal Al–Co–Ni on the (110) surface of *cP*2-Al(Co,Ni) (B2 or β phase) (Steurer, 2006*b* ▸) or of icosahedral Ti–Ni–Zr on (0001)-Al_2_O_3_ (Willmott *et al.*, 2005 ▸).

## 6. Conclusions   

What do we want to know about the structure of a periodic crystal? The answers are: the content of the unit cell, its structure and the kind of chemical bonding based on quantum-mechanical calculations. If the LRO is periodic, then there is no need to study it except in the case of intrinsic disorder. In the case of a quasiperiodic structure, however, both the SRO and the LRO need to be determined. In some cases this is only possible up to some limit, but this may still be sufficient to gain a good understanding. Our absolute limit, at present, is that we are not able to use quantum-mechanical calculations for QCs in the same way as for periodic crystals in order to identify when, for instance, it is advantageous for a structure to become quasiperiodic instead of forming a high-order AC.
